# Retrograde Cerebral Air Embolism Associated With Bronchovenous Fistula

**DOI:** 10.7759/cureus.34691

**Published:** 2023-02-06

**Authors:** Alla Uts, David Li, Daniel Kurbanov

**Affiliations:** 1 Internal Medicine, Mount Sinai South Nassau, Oceanside, USA; 2 Radiology, Mount Sinai South Nassau, Oceanside, USA; 3 Pulmonary and Critical Care Medicine, Mount Sinai South Nassau, Oceanside, USA

**Keywords:** lung cancer erosion venous circulation, retrograde air embolism, small cell lung cancer, bronchovenous fistula, cerebral air embolism

## Abstract

Cerebral air embolism is a rare event and predominantly iatrogenic. Here we present a case of spontaneous intravascular cerebral air embolism caused by lung cancer, which is among the other previously reported cases worldwide. A 69-year-old man with small cell lung carcinoma presented after being found unconscious. Computed tomography (CT) of the chest revealed a lung mass eroding into the superior vena cava (SVC) and with communication to the right upper lobe bronchus. As the patient’s neurologic status deteriorated further, serial CT scans of the brain noted multiple air emboli with development of left cerebral infarction, and death followed shortly after. This case highlights the rapid progression of this rare condition and thereby the need to be familiar with the clinical setting in which the presence of cerebral air embolism can occur.

## Introduction

Air embolisms are infrequent and potentially fatal and may occur as a complication of surgery, endoscopic procedures, vascular catheterization, trauma, or positive pressure ventilation. Air embolism occurs when there is both direct communication and a pressure gradient between air and the vasculature [1,2]. Most air embolisms are due to iatrogenic causes. Reported cases of air embolism in the setting of lung cancer are mainly due to complications of transthoracic needle biopsies, which are presumed to be via a creation of a bronchovenous fistula by the needle itself [3].

Cerebral air embolisms are even rarer presentations; there has been a total of only three reported cases of patients with lung cancer who developed cerebral air embolisms without any inciting factors of trauma or surgeries. Two cases were reported from Japan in men with non-small cell lung cancer [4,5], and most recently an additional case was reported from Poland involving suspected lung cancer with left-sided cardiac infiltration [6]. Overall prognoses and outcomes were poor, and all cases were reported to involve arterial air embolism by putative mechanism of air entry or radiographic characterization. We present a rare case of a 69-year-old man with aggressive lung cancer that infiltrated into the venous circulation and caused massive cerebral air embolism with ischemia.

## Case presentation

A 69-year-old man with a past medical history of small cell lung cancer, which was diagnosed two years prior, post chemotherapy and radiation therapy complicated by recent immunotherapy-induced pneumonitis, presented after his family found him unresponsive. The patient arrived on a bag valve mask, Glasgow Coma Score 3, with mild hypotension and tachycardia. He was immediately intubated for airway protection. On physical examination, bilateral horizontal nystagmus was noted with tracheal deviation to the right side and bilateral rhonchi.

**Figure 1:**
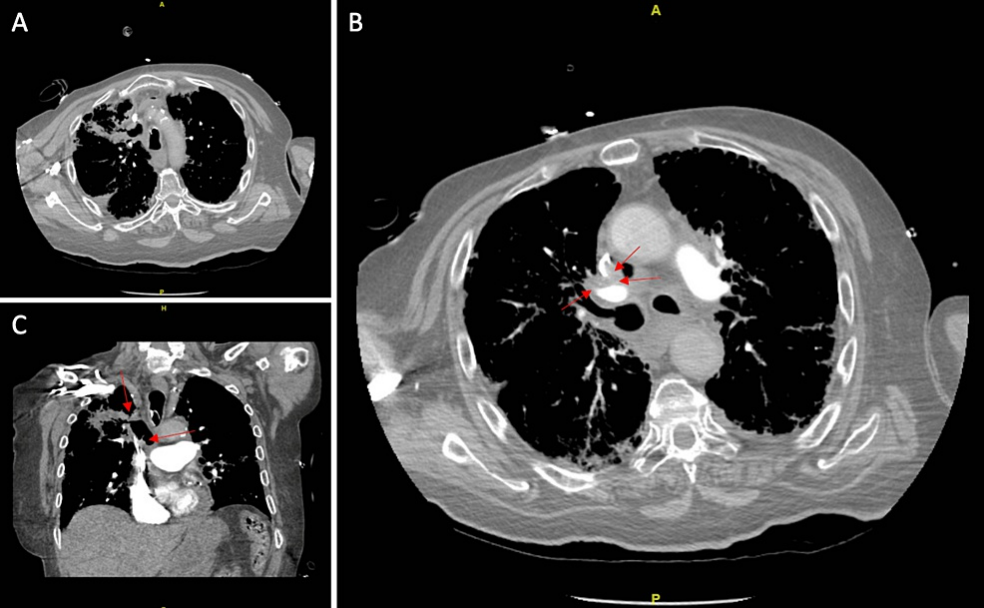
Contrast-enhanced axial CT images of the chest, timed with a CT pulmonary angiogram protocol. (A) Axial view of chest at the level of the aortic arch demonstrating cavitary mass with communication of the cavitary space with a right upper lobe bronchus. (B) Axial view of the chest at the level of the main pulmonary artery (inferior to A). Arrows demarcate soft tissue component of the mass which demonstrates intraluminal erosion into the superior vena cava. (C) Coronal view of the chest incorporating axial images A and B. Red arrows depict communication of the cavitary component of the mass to the bronchus and erosion of the mass into the SVC. Constellation of findings compatible with bronchovenous fistula from lung carcinoma.

Contrast-enhanced CT of the chest revealed a soft tissue density mass at the right upper lobe, consistent with the patient’s underlying history of small cell lung cancer, with encasement of the pulmonary artery and associated 3.9 cm cavitary space with communication to the right upper lobe bronchi. There was also noted soft tissue erosion of the mid aspect of the superior vena cava (SVC) (Figure 1, panels A-C).

**Figure 2:**
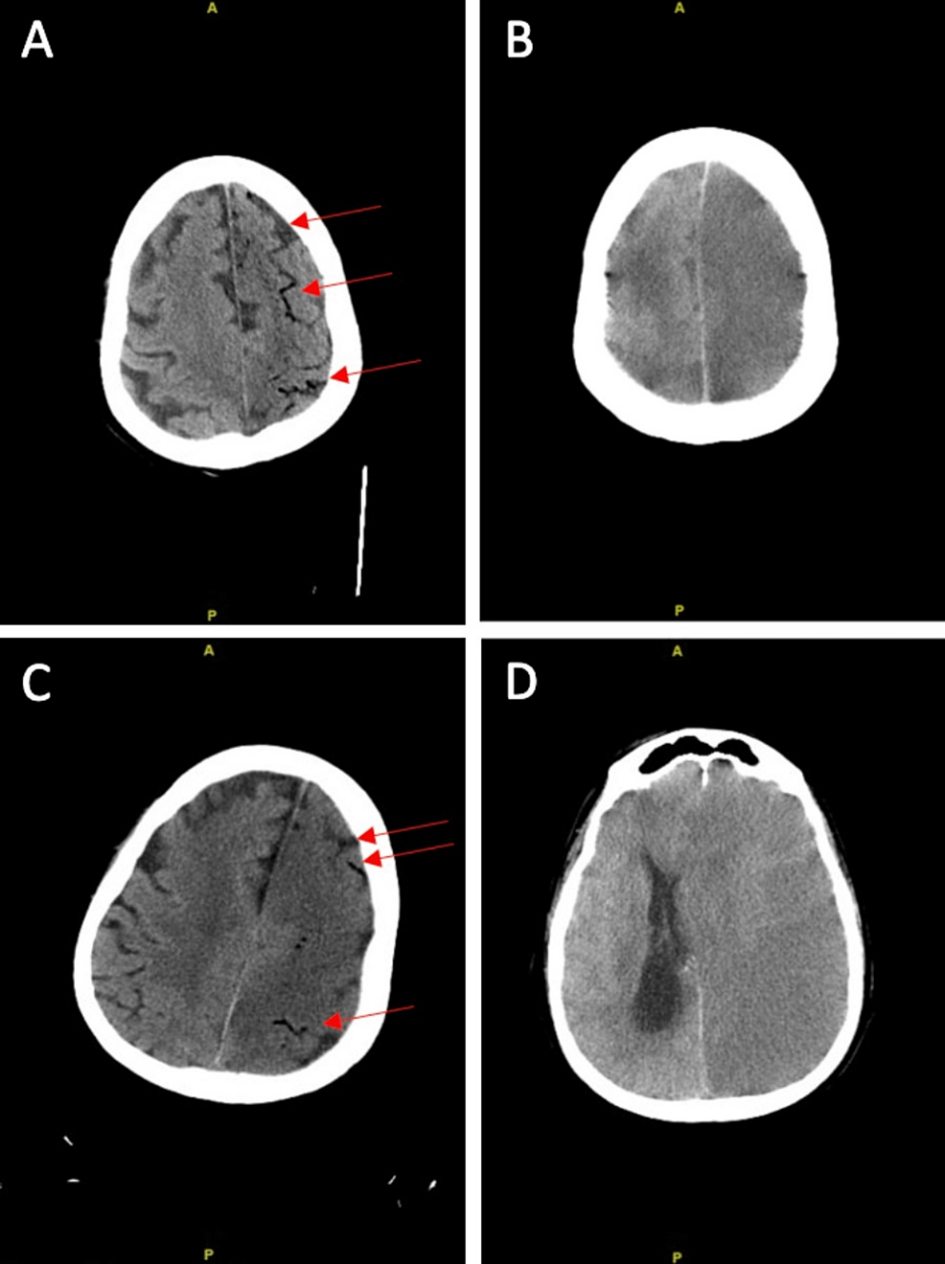
Non-contrast axial CT images of the brain over three consecutive days. (A) At initial presentation, red arrows highlighting multiple serpiginous low attenuation density foci in the frontal and parietal subarachnoid space. Differential diagnosis includes fat in the setting of ruptured dermoid versus air. (B) Twenty-four hours after initial presentation, resolution of low-density foci compatible with air dissipation, prompting diagnosis of air embolism. (C) Twenty-four hours after initial presentation, loss of normal grey-white matter differentiation involving the left hemisphere. Red arrows demonstrating persistent low-density foci compatible with air emboli. (D) Forty-eight hours after initial presentation, complete left cerebral hemisphere edema and sulcal effacement compatible with acute infarction at the level of the ventricles with associated rightward midline shift were seen.

**Figure 3:**
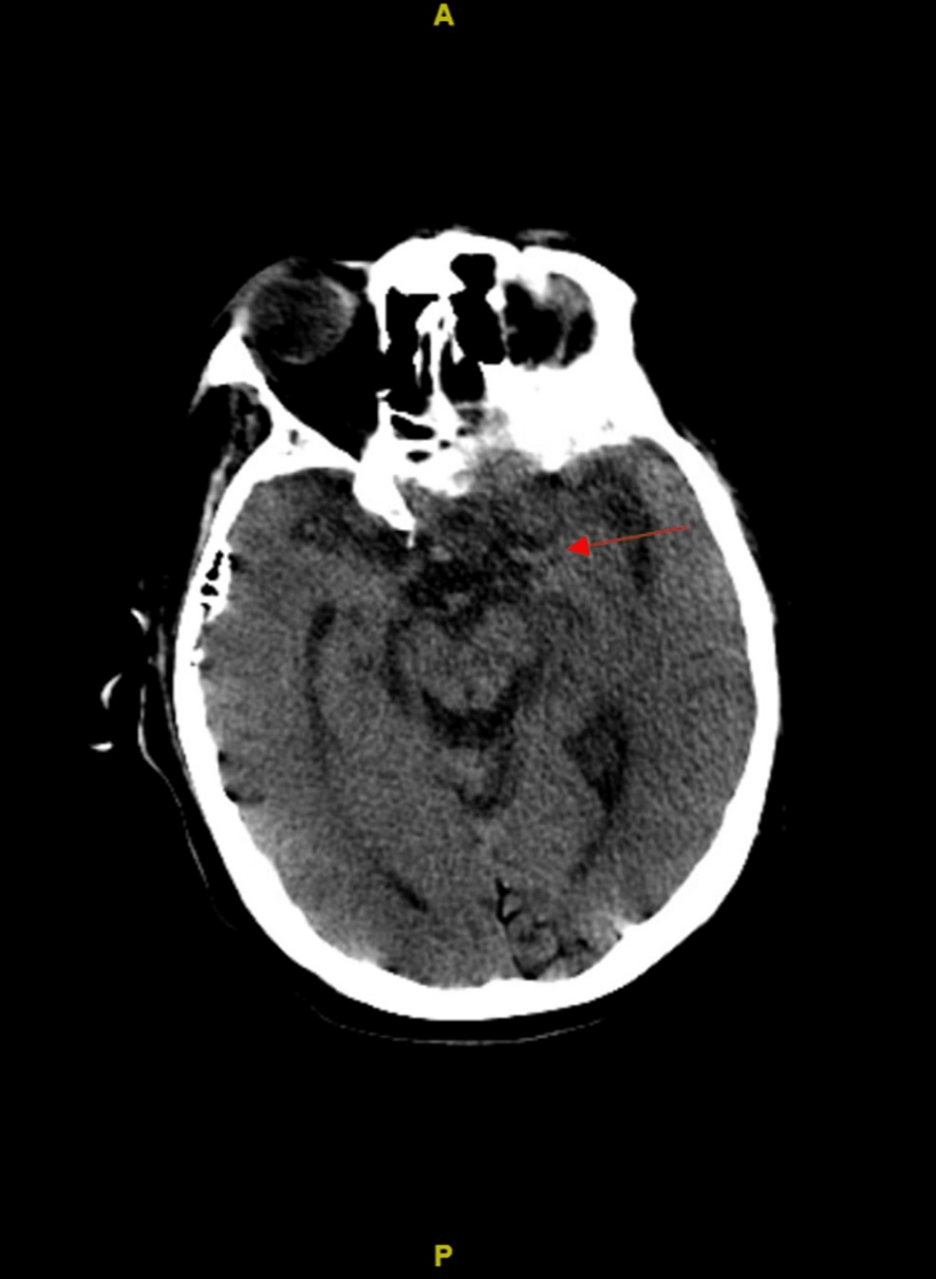
Non-contrast CT head axial image. Initial CT head without evidence of emboli in the middle cerebral artery highlighted by the red arrow.

Initial CT of the head revealed extensive serpiginous opacity of low-density overlying the left cerebral hemisphere in the subarachnoid space (Figure 2, panel A). Differential diagnosis included fat in the setting of ruptured dermoid versus air. On subsequent head CTs, there was resolution of the low-density opacity in the original area, prompting a diagnosis of air embolism (Figure 2, panel B). There was no evidence of embolism in the middle cerebral artery (Figure 3). CT of the head three hours following the initial scan revealed an acute large-volume left cerebral infarction with multiple air emboli throughout the left cerebral hemisphere (Figure 2, panel C). Forty-eight hours after the initial presentation, the patient was noted to have loss of brainstem reflexes and dilated pupils bilaterally (Figure 2, panel D). EEG demonstrated severe diffuse cerebral dysfunction, more prominent over the left hemisphere. There were no epileptiform potentials or seizures. CT of the head was repeated and revealed diffuse severe left cerebral edema, a new 1.7 cm of left-to-right midline shift, and effacement of the cisternal spaces indicative of subfalcine and uncal herniations. Neurosurgical intervention was determined to be non-beneficial. The patient’s hemodynamic instability progressed further and ultimately led to his demise a few hours after the last CT scan. MRI brain and transesophageal echocardiogram were not able to be obtained due to the patient’s rapid deterioration.

## Discussion

In the case presented, the patient developed spontaneous air embolism causing massive cerebrovascular infarction. The likely cause is progression of lung carcinoma with the development of bronchovenous communication. The potential contribution of known recent checkpoint inhibitor pneumonitis is also worth considering. There have been two proposed mechanisms for the cause of cerebral air embolism. One is arterial embolism by direct communication or paradoxical embolism in the presence of a right-to-left shunt. The other is via retrograde movement from the venous system into cerebrovascular circulation [7]. We suspect the latter to be the most probable cause for our patient’s emboli.

We hypothesize that the emboli traveled in retrograde fashion causing it to scatter throughout the subarachnoid space. A unique feature of air embolus is its ability to travel against the normal direction of blood flow due to its lower specific gravity [8]. Despite not being able to evaluate for a right-to-left shunt, the absence of emboli in the middle cerebral artery supports venous origin. In veins above the level of the heart, such as the SVC, the atmospheric pressure may be higher than the circulatory pressure, which allows air to enter the bloodstream. In our patient, air entered the systemic circulation through the close communication of the advanced lung cancer with the SVC, causing a bronchovenous connection (Figure 1, panels A-C). From there it then rose to the cerebral venous circulation against the normal direction of flow. Once the air entered the parenchyma, neutrophils were activated, and stasis was achieved, postulating the creation of a subsequent infarction versus thrombosis [2].

## Conclusions

We review a rare case of a patient with aggressive lung cancer who developed cerebral air emboli as a result of mass infiltration into bronchus and SVC. Given the fast progression of the disease, the medical community should be familiar with the risk of expanding lung cancer and its potential to lead to neurologic catastrophe.
